# Modelling Contaminant Formation during Thermal Processing of Sea Buckthorn Purée

**DOI:** 10.3390/molecules24081571

**Published:** 2019-04-20

**Authors:** Oana Emilia Constantin, Kristina Kukurová, Ľubomír Daško, Nicoleta Stănciuc, Zuzana Ciesarová, Constantin Croitoru, Gabriela Râpeanu

**Affiliations:** 1 Integrated Center for Research, Expertise and Technological Transfer in Food Industry, Faculty of Food Science and Engineering, Dunarea de Jos University of Galati, 111 Domnească Street, 800201 Galati, Romania; econstantin@ugal.ro (O.E.C.); nicoleta.stanciu@ugal.ro (N.S.); 2 VUP Food Research Institute, National Agricultural and Food Centre, Department of Chemistry and Food Analysis, Priemyslená 4, 82475 Bratislava, Slovakia; kukurova@vup.sk (K.K.); dasko@vup.sk (L.D.); ciesarova@vup.sk (Z.C.); 3 Academy of Agricultural and Forestry Sciences Gheorghe Ionescu-Sisesti, 61 Marasti Blvd, 011464 Bucharest, Romania; c.croitoru@sodinal.com

**Keywords:** acrylamide, hydroxymethylfurfural, sea buckthorn, temperature, time, thermally treatment, modelling design

## Abstract

*Background*: The impact of thermal treatment on acrylamide (ACR) and hydroxymethylfurfural (HMF) formation was investigated for thermally treated sea buckthorn purée. *Methods*: An optimized procedure for minimizing ACR and HMF formation in thermally treated sea buckthorn purée was described. The precursors of ACR and HMF and their impact in heating of sea buckthorn purée to obtain jam-like products were also evaluated. *Results*: The contaminant content formed in samples was analyzed on thirteen running variants using a temperature range of 59.3–200.7 °C, and for heating durations between 5.9 and 34.1 min. The calculated equations of contaminant formation in sea buckthorn purée have established that the minimum content is formed at the lowest exposure time, between 10 and 20 min, for both ACR and HMF. The lowest ACR content was attained at 5.9-min exposure time and 130 °C temperature (0.3 µg/kg). For HMF the results revealed a lower quantity at 59.3 °C for 20-min exposure time (1.4 mg/kg). Conclusions: the found model is useful for the prediction of the best temperature/time conditions of the thermal treatment to obtain the lowest contaminates levels in the final product.

## 1. Introduction

Sea buckthorn is a plant native of Europe and Asia [1]. Different parts of sea buckthorn have been used in herbal medicine to treat various diseases (e.g., influenza, mucosal and skin injuries) [2,3] and sea buckthorn parts are known as an important source of bioactive compounds such as carotenoids, tocopherols, vitamins, minerals [1,4]. In the food industry, although the sea buckthorn berries have a sour taste, they are used to make jams and jellies [5] alone or in combination with other fruits in varying amounts in order to attenuate the pungent taste [6]. The sea buckthorn berries are also used as purées for obtaining nectars, ice creams, baby food, dessert sauces, or can be added in food products as a dried powder.

For the thermally treated products with a high sugar and asparagine contents, at some stage during the production process, the Maillard reaction can occur, as highlighted by color [5] and flavor changes [7] and reductions in the nutritional food value [8]. Furthermore, a high temperature (above 120 °C), combined with sugar contents and the presence of amino acids, may lead to the formation of contaminants such as ACR and HMF [9,10,11]. The potential carcinogenicity of ACR has not been demonstrated in humans, but is well known that ACR has neurotoxic and carcinogenic potential in animals. Acrylamide can be found in products that were subjected to heating, such as baked potato products [12], baking and pastry products [13,14], jams [15,16], baby food [17,18], dried fruits [16,19], coffee [20,21], etc. It has been demonstrated that the HMF at high concentrations has a negative influence on humans, such as mucous membrane and skin cell cytotoxicity [22].

The HMF content of food products can be used as an indicator of the intensity of heat treatment during the production or storage of food products under improper conditions. HMF can be found in jams [5], fruit juices [23], baby food [15], dried fruit [24,25] and honey [22,26].

Of our knowledge, there is no information about the formation/reduction of acrylamide and HMF in thermally treated sea buckthorn fruits. The existing studies have been focused on the optimization of potato and cereal products as well as the control of the baking temperature. Therefore, the main purpose of this study was to identify the optimal time/temperature sequence to reduce 5-HMF and ACR formation during thermal treatment of sea buckthorn purée, which is the basic treatment for obtaining sea buckthorn puree and jams. The study intends to help manufacturers in establishing a procedure with minimal effect on the final product regarding the presence of HMF and ACR content.

## 2. Results

### 2.1. Determination of ACR and HMF Precursors and Antioxidant Compounds

The sea buckthorn berries were analyzed for neocontaminant precursors (amino acid content and reducing sugars); the results obtained are shown in Table 1. 

The chemical profile regarding the precursor composition of sea buckthorn is variable, depending on soil, cultivar, habitat and climatic conditions, and also the extraction conditions. The amino acid profile revealed the presence of 19 amino acids in sea buckthorn purée, from which six of those identified (leucine, isoleucine, methionine, phenylalanine, tryptophan, valine) are essential for organisms and two are involved in ACR formation (asparagine and aspartic acid). As it is known, asparagine is the amino acid responsible for the direct production of ACR, being the principal precursor [27]. Sea buckthorn raw purée presented a significant amount of free amino acids, where the asparagine content was 194.55 ± 5.00 mg/kg DW. Therefore, asparagine is quantitatively, by far, the most important, regarding the involvement in ACR formation. Moreover, another pathway for ACR formation is the reaction between ammonia and acrylic acid starting from aspartic acid, β-alanine, carnosine, and cysteine as precursors [10,27]. The content of aspartic acid and β-alanine in sea buckthorn raw purée was 5.25 ± 0.02 mg/kg DW and 5.09 ± 0.20 mg/kg DW, respectively. Furthermore, other amino acids may have a positive effect in reducing ACR in some model systems, such as proline, tryptophan, cysteine, glycine, lysine [28,29].

The total reducing sugar content in sea buckthorn purée was 2.86±1.03 mg/g DW (Table 1). Characterization of sea buckthorn juice regarding the total sugars, as a sum of glucose and fructose, was made by Tiitinen et al. [30], who revealed a variation between 1.9 and 7.1g/100 mL of juice as a function of pedoclimatic conditions and extraction technology. Sea buckthorn purée revealed a TPC of 66.67 ± 3.74 mg/g DW and an AA of 46.1 ± 2.2 mmol TE/g DW. The results are by those reported by Korekar et al. [31], varying from 9.64 to 107.04 mg GAE/g DW. For AA, Kumar et al. [32] reported values ranging from 86.35 to 343.86 mg TE/g. Several studies have revealed that the phenolic compounds and plant extracts may influence positively or negatively the ACR formation [33,34,35,36].

### 2.2. Contaminants (ACR and HMF) Formation

**Figure 1:**
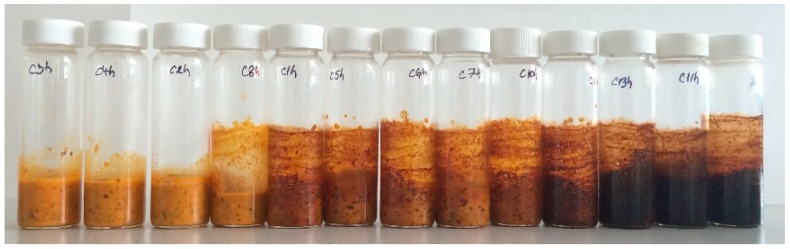
Sea buckthorn purée treated according to the experimental model.

The experimental conditions for HMF and ACR production for the present study were: temperature range between 59.3 and 200.7 °C and time between 5.9 and 34.1 min (Table 2, Figure 1).

For the estimation of the statistical parameters, ANOVA variance analysis was applied. The relevance of the model was also assessed assuming the multiple determination coefficient R^2^ (similar to the regression coefficient). As can be seen in Table 3, a low HMF content was obtained for variants 1, 11 and 13. The results revealed that at low temperatures used for the thermal treatment, the amount of HMF formed was lower, even if the treatment duration was longer. Similar results were obtained for the ACR content.

Optimized coding models for HMF and ACR content were represented by variance analysis and quadratic models applied. The values of *p* ≤ 0.05 show that the pattern is statistically significant. From the ANOVA table (Table 4) it can be seen that the models fit well for optimization data for both HMF (R^2^ = 0.9796) and ACR (R^2^ = 0.9700). The predicted R^2^ of 0.8561 for HMF was in reasonable agreement with the adjusted R^2^ of 0.9650. Similar data were obtained for ACR, where the predicted R^2^ of 0.7870 was also in reasonable agreement with the adjusted R^2^ of 0.9487.

For HMF and ACR the model F-values of 67.18, and 45.34 respectively, implying that the pattern was significant. The Prob > F values less than 0.05 indicated that model terms are also significant. The HMF content was positively correlated with all individual terms, with the greatest influence of the temperature (A) and quadric temperature (A^2^) response terms. For ACR, the relevant model terms were A, B, AB, and A^2^.

**Figure 2:**
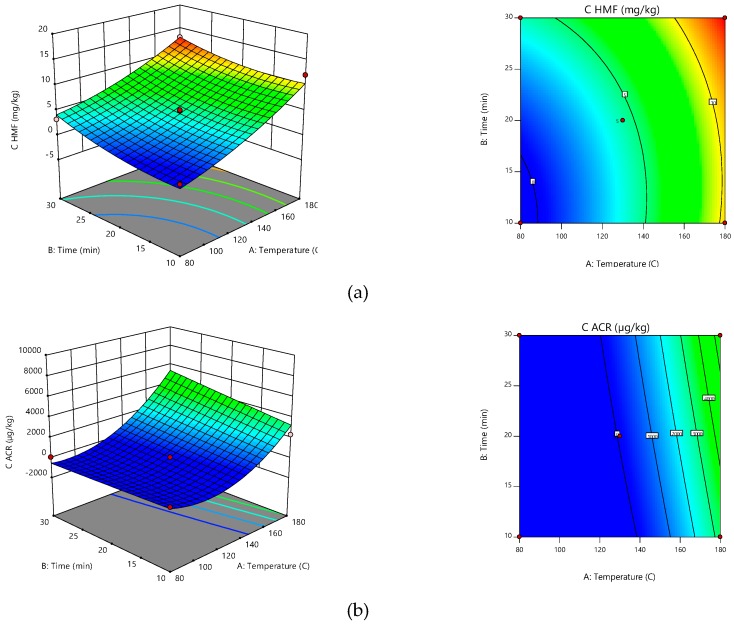
Response surfaces (right) and contour graphs (left) for defining the time and temperature correlative effects on the HMF (**a**) and ACR (**b**) formation.

Figure 2a,b revealed in the correlated temperature-time effect on HMF and ACR production. HMF formation was minimal at an exposure time, between 15 and 20 min, and at an exposure temperature of around 90 °C. ACR formation is minimal at an exposure time, between 15 and 20 min, and at an exposure temperature of around 130 °C.

Equations and β coefficients showing the models suitable for the production of ACR and HMF sea buckthorn samples are presented in Table 5. The equations in terms of coded factors showed that both time and temperature (A and B) have a positive effect on HMF and ACR production. This suggests that high values of temperature and long exposure time can lead to higher amounts of contaminants, which is in agreement with Figure 2a,b.

**Figure 3:**
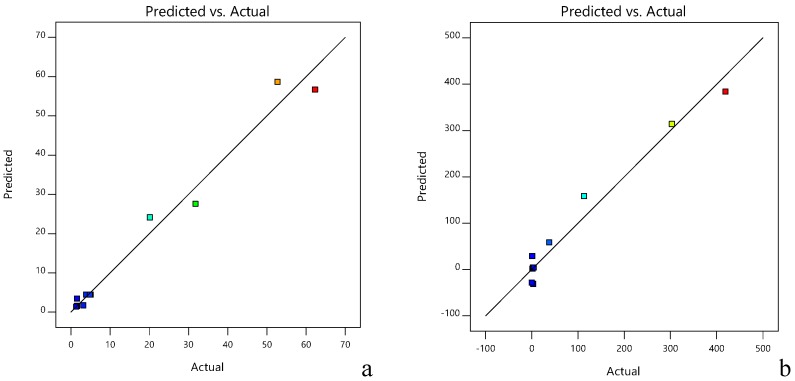
Predicted versus actual values for HMF (**a**) and ACR (**b**) content of sea buckthorn.

The comparison between HMF and ACR contents found experimentally (current values) and predicted, demonstrated the fidelity of the model (Figure 3). The grouping of the experimental values and those calculated by the model near the regression line shows that the chosen model is appropriate.

### 2.3. Global Optimization

**Figure 4:**
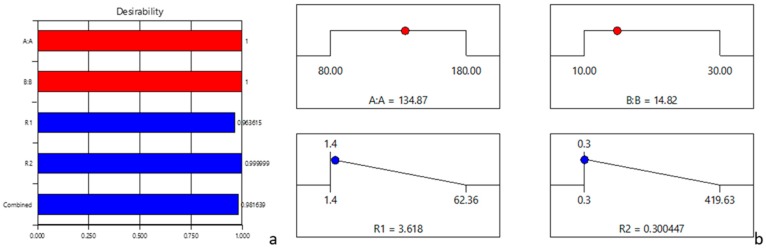
Chart of individual desirability functions. (**a**)—desirability chart; (**b**)—desirability solution; AA—temperature; BB—time; R1—HMF; R2—ACR.

The desirability values and the predicted responses at different levels of each factor were calculated. As the temperature decreased, the ACR and HMF levels also decreased and approaching to the minimum values (3.62 and 0.30 respectively). Figure 4 shows the desirability solution separately and combined into a single response. The desirability values obtained were of 0.96 for ACR and 0.99 for HMF respectively (Figure 4a). A value near 1 (0.98) displayed the right value in combinations. A non-zero value of desirability suggested that all the selected conditions were in a suitable combination.

The optimization objective was to obtain also a good set of conditions to ensure the lowest content for ACR and HMF by adjusting the thermal treatment process. Numerical optimization ramps shown in Figure 4b described a graphical representation of optimal solutions for the models chosen: the optimal factor settings were shown with red points (134.87 °C/14.82 min), and the optimal response prediction values were displayed in blue (HMF—3.618 mg/kg DW and ACR—0.300447 µg/kg DW).

## 3. Discussion

In the food industry, the Maillard reaction is used for its positive effect in food products such as color modification, texture improvement, and flavor formation, for enhancing the food palatability [37] of baked food [38,39], cocoa and coffee [40]. Despite the positive effects, the Maillard reaction can also cause negative impact in food, ranging from texture, flavor, and color changes to the formation of potentially toxic compounds with negative health effects (ACR, HMF, furans, and heterocyclic amines). Information about ACR formation in thermally treated sea buckthorn purée (such as jams) are missing in the literature. Therefore, the model adopted in this study was intended to be a strategy to limit the formation of potentially toxic compounds (ACR and HMF) in sea buckthorn purée by choosing the optimal combination of time/temperature. The high level of asparagine (194.55 ± 5.00 mg/kg DW) can explain the levels of ACR in the sea buckthorn purée treated at a temperature of 200 °C, mainly because is already known that the main carbon source for ACR formation is asparagine [41]. Asparagine decarboxylation and deamination by heating without the presence of sugar can lead to ACR formation, but the conversion to ACR depends on sugar presence [27,42]. Therefore, the presence of asparagine in the food matrix is important, and the amount of reducing sugars is critical in ACR formation [9,10,27,43]. The ACR formation in these types of food matrix may also be influenced by the presence of antioxidant compounds such as polyphenols, and vitamins. According to Liu et al. [44], the presence of gallic acid influenced the ACR elimination negatively by scavenging free radicals that can lead to the theory that the presence of polyphenols may increase the ACR content. However, in another study, Zhu et al. [35] reported that plant extracts in an asparagine-glucose system significantly reduce the ACR formation of ACR, by 69–75%.

The HMF levels within the model tested reached values between 1.4 and 62.36 mg/kg. Rada-Mendoza et al. [15] obtained from different types of fruit jams a variable content of HMF (5.5–37.7 mg/kg). In a study conducted by Aslanova et al. [5] regarding the HMF content of imported commercial jams (strawberry, cherry, and apricot) the initial values of HMF were 20.39, 34.18 and 30.83 mg/kg, respectively. In a study which examined the physicochemical changes of grape must heated at 95 °C/18 h for obtaining concentrated musts of 35%, 60%, and 70%, the HMF levels were also monitored [45]. The high levels obtained (216–592 mg/L) can be explained by the important precursor concentrations (amino acids, glucose, and fructose) from the thermally concentrated must. Although the HMF formation is usually related to reducing sugar using as basic substrate monosaccharides (fructose or glucose) [46], the amino acids significantly influenced the HMF content [47]. Lee and Nagy [48] showed amino acids increase the HMF content probably by sucrose hydrolysis. Furthermore, by heating fructose in the presence of aspartic acid at a pH of 7.0 is leading to a higher content of HMF, compared with the thermal treatment of fructose exclusively [49].

For sea buckthorn thermally treated products the regulatory bodies have not set so far a limit for ACR and HMF content. Moreover, the daily intake of ACR and HMF is usually influenced by individual consumption-patterns. However, the food-industry environment consistently pursued measures to reduce the amount of ACR and HMF in food products by applying effective quantification strategies [28,29,33,34,35].

## 4. Materials and Methods

### 4.1. Reagents and Chemicals

ABTS [2,2-azino-bis(3-ethylbenzothiazoline-6-sulphonic acid)], DNS (3,5-dinitrosalicylic acid), HMF standard (purity 99%) and 3,4,5-trihydroxybenzoic acid (gallic acid) were obtained from Sigma Chemical Co. (St. Louis, MO, USA). 6-Hydroxy-2,5,7,8-tetramethylchromane-2-carboxylic acid (Trolox), Folin-Ciocalteu reagent, potassium persulfate (K_2_S_2_O_8_), sodium carbonate, and ethanol were obtained from Sigma Aldrich (St. Louis, MO, USA). Acrylamide, purity 99% (Sigma-Aldrich), d3-acrylamide (2,2,3-d3-2-propenamide—d3-ACR), d3-glutamic acid (Cambridge Isotope Laboratories, Andover, MD, USA). 4-*trans*-hydroxyproline (Hyp), alanine (Ala), arginine (Arg), asparagine (Asn), aspartic acid (Asp), glutamic acid (Glu), glutamine (Gln), glycine (Gly), histidine (His), isoleucine (Ile), leucine (Leu), lysine (Lys), methionine (Met), ornithine (Orn), phenylalanine (Phe), proline (Pro), serine (Ser), threonine (Thr), tryptophan (Trp), tyrosine (Tyr), valine (Val) were provided by Sigma-Aldrich (Steinheim, Germany). Ethyl acetate, phosphoric acid (H_3_PO_4_), acetonitrile, acetic acid, methanol HPLC-grade were all purchased from Merck (Schuchardt, Germany). Syringe filters (nylon, 0.45 μm) were obtained from Waters (Milford, MA, USA).

### 4.2. Samples

Fully ripe sea buckthorn berries (*Hippophae rhamnoides* L.) Botanicky cultivar has been collected from the local market (Bratislava, Slovakia) and stored at 4 °C before analysis. Berries were washed, homogenized at 10,000 rpm/15s (Grindomix GM 200, Retsch, Haan, Germany), and heat treated according to the experimental model using a thermostat (Liebisch Labortechnik, city, Germany).

### 4.3. Determination of Total Phenolic (TPC) and Antioxidant Activity (AA)

Freeze-dried berries were ground in a mortar and were weighted, and phenolic constituents were twice extracted in a ratio 1:5 (*w*/*w*) with the ethanol (70%) in a thermostatic rotary at a temperature of 28 °C for 2 h. The liquid extracts were separated from solids by centrifugation (11800 g for 15 min, Rotanta 460 R centrifuge, Hettrich, Tuttlingen, Germany) and the supernatants were combined. TPC and AA of the extract were valued as reported earlier by Constantin et al. [50]. For TPC determination, a modified Folin-Ciocalteu method was applied. Briefly, 200 µL extract was mixed with 125 µL Folin-Ciocalteu reagent and was stirred for 3 min. In the mixture of a 125 µL Na_2_CO_3_ 20% and 550 µL distillate water, were added. The mixture was allowed to stand for 30 min at the room temperature. In the end, the mixture was centrifuged for 10 min at 8200 g, and the absorbance of the final solution was measured at 765 nm. The results were expressed as gallic acid equivalent (mg GAE/g DW). Antioxidant activity was achieved using the ABTS + radical test (AA). The extracts were mixed with an ABTS + solution (diluted from a 7 mM stock solution with 2.45 mM K_2_S_2_O_8_), in order to react. Then the absorbance was measured at 734 nm. The results were expressed in mmol Trolox/g DW.

### 4.4. Acrylamide and HMF Precursors

The amino acids extraction from sea buckthorn berries was performed as reported earlier by Constantin et al. [16]. Determination of reducing sugars was performed by the DNS method (3,5-dinitrosalicylic acid) according to A.O.A.C. [51].

### 4.5. Acrylamide and HMF Content

After heat treatments of sea buckthorn purée, the acrylamide (ACR) was extracted with 0.1% acetic acid and further pre-extracted to ethyl acetate and quantified by LC-MS analysis according to Constantin et al. [52], using a LC/ESI-MS-MS HPLC Agilent 1260 Infinity system coupled to 6410 Triple Quadrupole LC/MS with ESI interface (Agilent Technologies, Santa Clara, CA, USA). A dC18 column (Waters, Milford, MA, USA) was used, and the elution gradient used a flow rate of 0.4 mL/min at 25 °C. The mobile phase consisted of methanol 1% and 0.2% acetic acid. The electrospray ionization—mass spectrometry (ESI-MS-MS) parameters All parameters of the electrospray ionization tandem mass spectrometry (ESI-MS-MS) were established on ACR, and the internal standard (d3-ACR) protonated molecular ions generation. The calibration was obtained starting from a stock solution of ACR (5 mg in 100 mL of water) obtaining values in the range of 10–2000 ng/10 mL by diluting with 50 µL of the internal standard (d3-ACR).

Concentrations of HMF were determined and evaluated as described by Tobolková et al. [44], using an Agilent 1200 HPLC system (Agilent Technologies). A C18 SB column (Waters, Milford, MA, USA) was used, and the elution gradient used a flow rate of 0.8 mL/min at 25 °C. The mobile phase consisted of methanol, H_3_PO_4_, 0.01 M, and acetonitrile. The composition of mobile phase for HMF determination was: 0-1.5 min, 0–2% A, 100–95% B, 0–3% C; 1.5–2.1 min, 2% A, 95% B, 3% C; 2.1–3.0 min, 2–8% A, 95–86% B, 3–6% C; 3.0–11.0 min, 8% A, 86% B, 6% C; 11.0–11.5 min, 8–94% A, 86–0% B, 6% C; 11.5–20.0 min, 94% A, 0% B, 6% C; 20.0–20.1 min, 94–2% A, 0–95% B, 6–3% C; 20.1–30.0 min, 2% A, 95% B, 3% C. The detection of HMF was obtained at 280 nm and calculated using a calibration curve in the range of 0.05 to 1.0 µg/mL.

### 4.6. Thermal Treatment and Experimental Design

Sea buckthorn purée samples were placed in glass tubes and heat treated by using a temperature interval between of 59.3 and 200.7 °C, and a heating period range between 5.9 and 34.1 min, according to the experimental model presented in Table 3.

Design of experiments was performed as described earlier by Constantin et al. [52] using Central Composite Design (CCD) and response surface modeling to optimize the thermal treatment of sea buckthorn purée in order to identify the optimal time/temperature combination for minimizing HMF and ACR formation. The CCD model used with two factors and three levels builds a quadratic model for response variables. The experimental design CCD imposed 13 experimental variants. For all variables, the zero-coded central value was considered. The maximum and minimum ranges of the variables investigated in the experimental plan in actual and coded form are shown in Table 2. The experiments were performed in sequence imposed by the software in order to establish the external factors influence in the analysis. Formation of ACR and HMF were considered as dependent or response variables.

The experimental conditions can be explained by the equation obtained by the Design Expert software [1]:

R (1, 2) = b_0_ + b_1_·A + b_2_·B + b_3_·A·B + b_4_ A^2^ + b_5_·B^2^ (1)

where A, B are independent variables; b_0_—intercept, b_1_—b_5_ regression coefficients.

The desirability approach was used as a tool to optimize processes with several response processes. The desirability function provides a mathematical solution when a diversity of measurements is linked to one precise indicator.

### 4.7. Statistical analysis

The multivariate data analysis and software Design Expert (v. 11) from Design-Expert^®^ (Stat-Ease, Inc., MN, USA) were used for data analyzing.

## 5. Conclusions

The contents of ACR and HMF were measured in thermally processed sea buckthorn purée, in order to optimize heat treatment to ensure a low content of contaminants. ACR formation was minimal at the shortest exposure time with the decrease in the amount of ACR formed as the temperature is reduced. The duration of the heat treatment is important for the HMF formation and squared values of the temperature also influences the formation of HMF in buckthorn purée. The study developed an optimized procedure for reducing ACR and HMF formation in thermally treated sea buckthorn purée by identifying the ideal combination of time and temperature. The optimized variants detected the following parameters (temperature/time) that should be applied for minimaxing the contaminants tested: 134.87 °C/14.82 min.

## Figures and Tables

**Table 1:** The precursor levels and antioxidative compounds in sea buckthorn berries.

Precursors				
**Amino acids**, mg/kg DW	Hyp	ND	Met	0.12 ± 0.00
	Asp	5.25 ± 0.02	Tyr	ND
	Pro	0.45 ± 0.02	Ile	0.61 ± 0.02
	Asn	194.55 ± 5.00	Leu	0.61 ± 0.02
	Ser	2.34 ± 0.08	Phe	1.50 ± 0.05
	Gln	7.44 ± 0.08	His	0.64 ± 0.02
	Thr	0.59 ± 0.03	Orn	0.23 ± 0.01
	Glu	1.43 ± 0.04	Lys	1.08 ± 0.03
	Gly	0.38 ± 0.02	Arg	5.01 ± 0.01
	Ala	5.09 ± 0.20	Trp	0.45 ± 0.01
	Val	0.54 ± 0.01		
**Reducing sugars**, mg/g DW	2.86 ± 1.03			
**Antioxidative compounds**				
**TPC**, mg/g DW	66.67 ± 3.74			
**AA**, mmol TE/g DW	46.1 ± 2.2			

4-trans-Hydroxyproline (Hyp), Aspartic acid (Asp), Proline (Pro), Asparagine (Asn), Serine (Ser), Glutamine (Gln), Threonine (Thr), Glutamic acid (Glu), Glycine (Gly), Alanine (Ala), Valine (Val), Methionine (Met), Tyrosine (Tyr), Isoleucine (Ile), Leucine (Leu), Phenylalanine (Phe), Histidine (His), Ornithine (Orn), Lysine (Lys), Arginine (Arg), Tryptophan (Trp). TPC is expressed as grams of gallic acid equivalents; AA is expressed as mmol Trolox equivalent/g DW. ND—Not detected.

**Table 2:** The factors studied and uncoded and coded levels used in the RSM design.

Factor	Name	Units	Variation Levels					
			Minimum	Maximum	Coded Low (−1)	Coded High (+1)	Mean	Std. Dev.
A	Temperature	°C	59.29	200.71	80.00	180.00	130.00	40.82
B	Time	min	5.86	34.14	10.00	30.00	20.00	8.16

**Table 3:** Experimental data for the with responses.

Run	Factor 1	Factor 2	Response 1	Response 2
	A: Temperature, °C	B: Time, min	HMF, mg/kg DW	ACR, µg/kg DW
1	130.00	5.86	1.58	0.30
2	180.00	10.00	31.80	113.64
3	130.00	20.00	5.09	3.96
4	130.00	20.00	3.94	3.91
5	200.71	20.00	52.78	419.63
6	130.00	20.00	4.91	4.61
7	130.00	20.00	3.93	3.30
8	130.00	34.14	20.18	38.14
9	80.00	30.00	3.22	3.59
10	130.00	20.00	4.25	3.94
11	80.00	10.00	1.6	1.98
12	180.00	30.00	62.36	303.70
13	59.29	20.00	1.4	1.31

**Table 4:** Analysis of variance for response surface quadratic model.

Response	Source	Sum of Squares	df	Mean Square	F Value	*p*-Value Prob > F
**HMF**	Model	5116.26	5	1023.25	67.18	<0.0001
	A	3280.59	1	3280.59	215.37	<0.0001
	B	427.55	1	427.55	28.07	0.0011
	AB	209.38	1	209.38	13.75	0.0076
	A^2^	1134.95	1	1134.95	74.51	<0.0001
	B^2^	151.58	1	151.58	9.95	0.0160
	Residual	106.63	7	15.23		
	*Lack of Fit*	105.44	3	35.15	118.31	0.0002
	*Pure Error*	1.19	4	0.30		
	Cor Total	5222.88	12			
	**R^2^**	**0.9796**	**Adj R^2^**	**0.9650**	**Pred R^2^**	**0.8561**
**ACR**	Model	2.136E + 005	5	42721.52	45.34	<0.0001
	A	1.258E+005	1	1.258E+005	133.57	<0.0001
	B	7514.39	1	7514.39	7.98	0.0256
	AB	8878.35	1	8878.35	9.42	0.0181
	A^2^	70940.47	1	70940.47	75.30	<0.0001
	B^2^	199.76	1	199.76	0.21	0.6592
	Residual	6595.08	7	942.15		
	*Lack of Fit*	6594.22	3	2198.07	10226.93	<0.0001
	*Pure Error*	0.86	4	0.21		
	Cor Total	2.202E + 005	12			
	**R^2^**	**0.9700**	**Adj R^2^**	**0.9487**	**Pred R^2^**	**0.7870**

**Table 5:** The β coefficients and the final equation in terms of coded factors for the model chosen.

Factors	β Coefficients	
	HMF (R1)	ACR (R2)
A	4.42	3.94
B	20.25	125.42
AB	7.31	30.65
A^2^	7.23	47.11
B^2^	12.77	100.98
**Final Equation in Terms of Coded Factors**	R1 = +4.42 + 20.25·A + 7.31·B + 7.23·A·B + 12.77·A^2^ + 4.67·B^2^	R2 = +3.94 + 125.42·A + 30.65·B + 47.11·A·B + 100.98·A^2^ + 5.36·B^2^

R1-Response 1; R2-Response 2.
